# Bacterial community distribution and functional potentials provide key insights into their role in the ecosystem functioning of a retreating Eastern Himalayan glacier

**DOI:** 10.1093/femsec/fiae012

**Published:** 2024-02-01

**Authors:** Srijana Mukhia, Anil Kumar, Rakshak Kumar

**Affiliations:** Biotechnology Division, CSIR – Institute of Himalayan Bioresource Technology, Post Box No. 06, Palampur 176061, Himachal Pradesh, India; Department of Microbiology, Guru Nanak Dev University, Amritsar 143005, Punjab, India; Biotechnology Division, CSIR – Institute of Himalayan Bioresource Technology, Post Box No. 06, Palampur 176061, Himachal Pradesh, India; Academy of Scientific and Innovative Research (AcSIR), Ghaziabad 201002, India; Biotechnology Division, CSIR – Institute of Himalayan Bioresource Technology, Post Box No. 06, Palampur 176061, Himachal Pradesh, India; Academy of Scientific and Innovative Research (AcSIR), Ghaziabad 201002, India

**Keywords:** adaptation, bacterial ecology, East Rathong Glacier, MAGs, nutrient cycling

## Abstract

Himalayan glaciers are receding at an exceptional rate, perturbing the local biome and ecosystem processes. Understanding the microbial ecology of an exclusively microbe-driven biome provides insights into their contributions to the ecosystem functioning through biogeochemical fluxes. Here, we investigated the bacterial communities and their functional potential in the retreating East Rathong Glacier (ERG) of Sikkim Himalaya. Amplicon-based taxonomic classification revealed the dominance of the phyla Proteobacteria, Bacteroidota, and candidate Patescibacteria in the glacial sites. Further, eight good-quality metagenome-assembled genomes (MAGs) of Proteobacteria, Patescibacteria, Acidobacteriota, and Choloflexota retrieved from the metagenomes elucidated the microbial contributions to nutrient cycling. The ERG MAGs showed aerobic respiration as a primary metabolic feature, accompanied by carbon fixation and complex carbon degradation potentials. Pathways for nitrogen metabolism, chiefly dissimilatory nitrate reduction and denitrification, and a complete sulphur oxidation enzyme complex for sulphur metabolism were identified in the MAGs. We observed that DNA repair and oxidative stress response genes complemented with osmotic and periplasmic stress and protein chaperones were vital for adaptation against the intense radiation and stress conditions of the extreme Himalayan niche. Current findings elucidate the microbiome and associated functional potentials of a vulnerable glacier, emphasizing their significant ecological roles in a changing glacial ecosystem.

## Introduction

Ever since the past century, a constant rise in global temperature has resulted in climate change (Jansson and Hofmockel 2020). Reports estimate that global warming may exceed the level of 1.5°C or 2°C by the next decade (IPCC 2022). The Himalayan regions are particularly more vulnerable to climate change than the global mean (Bajracharya and Shrestha 2011). One major consequence of climate change is the shrinkage of glaciers, which directly impacts the downstream ecosystems as mountain glaciers are essential regulators of the global atmospheric, hydrological, and biogeochemical cycles (Milner et al. 2017). Studies on the impact of deglaciation have exclusively been focussed on macro-level dynamics, while research concerning its effect on glacial microbial communities is still at its nascency. The study of the glacier microbiome is significant as glaciers are recognized as unique biomes driven exclusively by microorganisms through global biogeochemical fluxes (Anesio and Laybourn-Parry 2012, Anesio et al. 2017). Microbial diversity holds a dynamic relationship with ecosystem functioning as they play multifunctional roles in primary production, nutrient cycling, organic decomposition, and climate regulation (Delgado-Baquerizo et al. 2016). Dominant microbial communities of the supraglacial surface are key drivers of the production and transport of dissolved organic carbon and nitrogen from the glacier to the downstream ecosystems for sustaining food webs (Milner et al. 2017). These microorganisms sequester the atmospheric labile inorganic nutrients, metabolizing them into an organic carbon pool and forming a hotspot for primary production (Anesio et al. 2009). Further, microbial colonizers are the pioneers of primary succession in deglaciated barren forefields, paving the way for plant and other complex community colonization (Ciccazzo et al. 2016). The expediting global temperature rise is accompanied by changes in precipitation and glacier coverage that are likely to alter the native microbial community and their functional roles in primary productivity and nutrient cycling in the glacier system (Rathore et al. 2022). These facts indicate that any loss of microbial diversity can lead to inefficient functioning of the terrestrial as well as aquatic ecosystems (Bodelier 2011, Cardinale et al. 2011).

While ecological and functional information on microbial life is widely covered from the glaciers and permafrost of Arctic and Antarctic regions (Edwards et al. 2013, Lutz et al. 2017, Li et al. 2019, Xue et al. 2020, Wu et al. 2021), Indian Himalayan glaciers are relatively less investigated. Some studies have been conducted in the glaciers of Indian Western Himalaya to gain insights into the microbial distribution patterns and their potential functional roles (Kumar et al. 2019, 2022, Bhattacharya et al. 2022, Rathore et al. 2022). Recent microbiome analysis of Changme Khang and Changme Khangpu in the Sikkim Himalaya has revealed a rich microbial diversity with scope for future biotechnological potential (Sherpa et al. 2019, 2020). Considering the significance of microbial diversity and the ecological roles they perform, the microbial community dynamics of the supraglacial and forefield regions of Sikkim Himalayan glaciers are rarely explored. A dominant reason for this lack of study is the remote and obscure location of the Himalayan glaciers, accompanied by rugged and rough hilly terrains (Rathore et al. 2022). The current research is conducted on one such high-altitude glacier in Sikkim, the East Rathong Glacier (ERG)—a benchmark glacier in the Eastern Himalaya for long-term monitoring of glacier mass balance, and hydrological balance owing to factors like ideal length, size, accessibility, and visible impact of climate change. The glacier retreated by 460 m within a timespan of 1980–2012 at an average rate of 13.3 m/year (Luitel et al. 2012). The present study aims to assess the microbial community composition and their functional potentials from the ablation zone supraglacial surface and the closest forefield site at an elevation gradient of 4600–4700 masl. We attempt to achieve a holistic view of the metabolic and stress adaptation strategies of the microbial communities, apprehending their significance in ecosystem functioning.

## Methods

### Study site and field sampling

The ERG is a south–east facing valley type glacier located between 88°06′27.63′′E and 27°34′54.44′′N within the Kanchenjunga National Park of Sikkim in the Eastern Himalaya (Mukhia et al. 2022a, Sharma et al. 2022). Covering an area of 4.80 km^2^ and ∼7 km in length, the glacier is debris-free and a source of the river Rangit. It takes a 39-km trek to reach the study site from the last accessible village, Yuksom, in West Sikkim.

**Figure 1.**
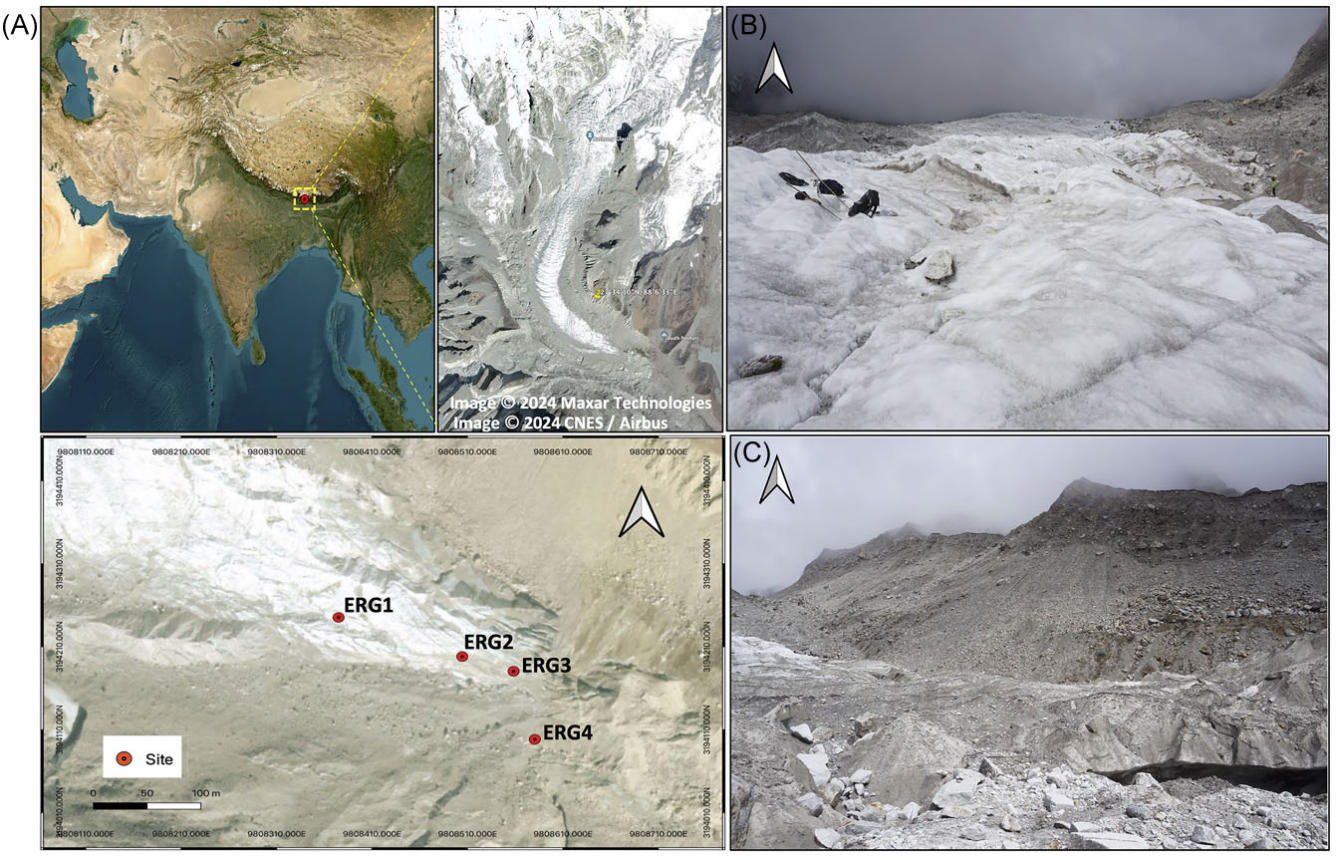
Location of the study area. (A) Map of sampling sites across the transect in the ERG, where ice-meltwater and sediment samples were collected in duplicates and triplicates, respectively, from each site. (B) Supraglacial site and, (C) Proglacial site. The map was generated in QGIS v3.26.2.

Sampling was conducted in pre-October 2021, encompassing the supraglacial and proglacial sites at an elevation of > 4600 masl. We designated the sampling sites as: Supraglacial site—ERG1 and ERG2, 4670 masl; Proglacial site—ERG3 and ERG4, 4648–4657 masl (Table 1 and Fig. 1). Specifically, proglacial sediment samples were obtained from bare and dry areas, whereas supraglacial samples were systematically collected along run-off channels. At the Supraglacial site, we collected ice-meltwater and sediment samples from two points (ERG1 and ERG2) on the glacier surface of the ablation zone. At the Proglacial site, soil sediments were collected from two points (ERG3 and ERG4) across the barren moraine in the immediate vicinity of the glacier snout. At each point, sediment sample collection was done in triplicates within a distance of ∼1 m, while the water sample was collected in duplicates. The ice meltwater sample (depth of 10–15 cm) was collected in a Sterivex filter unit (Millipore, USA), and the same was stored in 50 ml tubes (Tarsons, India) for physicochemical analyses, while sediment samples (depth of 5–10 cm) were collected in sterile sample bags. The samples were transited with ice packs till Yuksom and further to the laboratory within 48 h by air.

**Table 1.** Description of sampling sites and sample types at each site along with physicochemical parameters (average ± standard deviation) of each sample; masl: metres above sea level, TC: total carbon, TN: total nitrogen, NA: not applicable/not measured.

Parameters	Site I (Supraglacial site)		Site II (Proglacial site)	
Site	Glacier surface	Glacier surface	Moraine	Moraine
Sample code	ERG1	ERG2	ERG3	ERG4
GPS coordinates	N27°33.88′E88°6.6777′	N27°33.9025′E88°6.6078′	N27°33.8715′E88°6.7067′	N27°33.8323′E88°6.7187′
Elevation (masl)	4670	4670	4648	4653
Sample type	Ice-meltwater	Sediment	Sediment	Sediment
pH	6.58 ± 0.12	6.16 ± 0.08	6.38 ± 0.08	6.19 ± 0.02
Conductivity (µS)	6.14 ± 0.06	10.41 ± 1.4	11.43 ± 0.9	12.56 ± 0.9
TC (%)	NA	0.11 ± 0.01	0.12 ± 0.01	0.19 ± 0.005
TN (%)	NA	0	0	0.01 ± 0.005
P (ppm)	0.25 ± 0.001	0.47 ± 0.002	0.61 ± 0	0.56 ± 0.001
K (ppm)	0	1969.42 ± 29.3	2204.31 ± 39.6	1059.58 ± 49.8
Zn (ppm)	0	40.58 ± 0.9	26.72 ± 0.1	45 ± 0.32
Fe (ppm)	0	3886.47 ± 9.8	3863.64 ± 38.8	4174.44 ± 14.2
Cu (ppm)	0.06 ± 0.01	15.51 ± 0.6	14.12 ± 0.33	21.53 ± 0.32
Ca (ppm)	12.17 ± 2.1	77.84 ± 4.4	40.37 ± 0	53.18 ± 1.7
Mn (ppm)	0	145.77 ± 1.9	154.31 ± 1.5	216.32 ± 2.07
Mg (ppm)	4.70 ± 3.05	739.94 ± 2.3	719.49 ± 0.04	738.18 ± 10.6
Pb (ppm)	0	0	0	0
Ni (ppm)	0	0	0	0
Cr (ppm)	0	0	0	0
Cd (ppm)	0	0	0	0

### Physicochemical analyses of samples

The pH and electrical conductivity (EC) of samples were determined using a digital pH and EC meter (Eutech PC 450, Thermo Scientific, USA). The sediment pH and EC were measured by preparing suspensions of 1:2.5 (w/v) (Broadbent et al. 2021) and 1:5 (w/v) (Bockheim 2007) in deionized water, respectively. Sediment samples were oven-dried, sieved, and cleared of rocks and gravel for chemical analyses. The total carbon (TC), nitrogen (TN), and sulphur (S) contents of the solid samples were measured using the CHNS elemental analyzer (Vario MICRO cube, Elementar, Germany). The elemental composition of the solid and liquid samples was determined using an Atomic Absorption Spectrometer (Shimadzu AA-6300, Japan) after the extraction process following the hot aqua regia digestion method (Rice et al. 2017, Kumari et al. 2022).

### DNA extraction

Total DNA from the sediment and water samples was extracted using a FastDNA spin kit for soil (MP biomedicals, California, USA) according to the manufacturer’s guidelines with few modifications like bead-beating at maximum speed for 10 min and prolonged protein precipitation at 4°C to increase the yield. Extracted DNA was subjected to nanodrop (Thermofisher Scientific, USA) and agarose gel assessments before PCR amplification.

### Amplicon and shotgun sequencing

For microbiome analysis, amplicon sequencing of supraglacial and proglacial samples was performed for the V3–V4 region of the 16S rRNA gene using the primer pair V13F: – 5′ AGAGTTTGATGMTGGCTCAG 3′ and V13R: – 5′ TTACCGCGGCMGCSGGCAC 3′ (Saxena et al. 2021). The PCR conditions included: initial denaturation at 95°C, 25 cycles of denaturation at 95°C for 15 s, annealing at 60°C for 15 s, elongation at 72°C for 2 min, and a final extension at 72°C for 10 min, using 12.5 ng template DNA. The amplicons from each sample were purified with AMPure XP beads to remove unused primers. Sequencing libraries were prepared using the Nextera XT DNA Library Preparation kit (Illumina, USA), following the manufacturer’s instructions. Libraries were purified using AMPure XP beads and quantified using a Qubit dsDNA high-sensitivity assay kit. Sequencing was performed on the Illumina MiSeq platform (Illumina).

To analyze the functional potential of the microbiota, shotgun sequencing was performed on two soil sediment samples (one each from the supraglacial and proglacial sites). Metagenome libraries were prepared using the KAPA HyperPlus kit (KAPA Biosystems, USA), following the manufacturer’s instructions with 400 ng template DNA. The DNA was fragmented using the KAPA fragmentation method into 600 bp length fragments. The fragmented samples were processed for end repair and A-tailing with the HyperPrep/HyperPlus ERAT enzyme mix. Immediately after the end repair and A-tailing, adapter ligation to the end-repaired DNA fragments using DNA ligase was performed. Postligation cleanup was performed using 0.7X AMPure XP beads to remove any unincorporated adapter. Library amplification of the adapter-ligated DNA samples was done using Illumina primers. Sequencing was performed using the Illumina Hiseq 4000 platform (Illumina).

### Data processing and taxonomic assignments

The raw reads generated from amplicon sequencing were demultiplexed to remove the barcodes using an in-house script. Quality check of the reads was done using FastQC v0.11.9 (https://www.bioinformatics.babraham.ac.uk/projects/fastqc/). Only forward reads were considered due to the suboptimal quality of the reverse reads. The adapters, primers, and sequence reads with a phred score < 30 were removed using Cutadapt v3.4 (Martin 2011). Further trimmed reads were analyzed using the QIIME2 v2021.2 pipeline (Bolyen et al. 2019). The denoising and chimera removal from the imported single-end reads were achieved using the DADA2 pipeline (Callahan et al. 2016) (Table S1, Supporting Information). For taxonomic assignments, we used the SILVA v138 database (McDonald et al. 2012) with q2-feature-classifier (Bokulich et al. 2018). The feature table and feature sequences were filtered using ‘qiime taxa filter-table’ and ‘qiime taxa filter-seqs’ commands for removing unassigned amplicon sequence variants (ASVs) and those annotated as mitochondria. The prefiltered, rarefied ASVs table and taxonomy were used for the calculation of Alpha diversity indices (Shannon index, Simpson index, and species observed) using the Phyloseq R package v1.46.0 (McMurdie and Holmes 2013). Additionally, the effect of different environmental factors on the microbial abundance of ERG was evaluated by redundancy analysis (RDA). The RDA was performed by employing microeco R package v1.2.0, which uses the rda function of the vegan package (Oksanen et al. 2019). To test the significant differences in the bacterial diversity between the sample groups, a Kruskal–Wallis Rank Sum test was performed at a 95% significance level, using the function Kruskal test in R v4.2. The workflow is provided in Table S2 (Supporting Information).

### Metagenome binning and analysis

The raw metagenomic reads were processed using the KBase server (Arkin et al. 2018) according to a previously described method (Chivian et al. 2022). The raw reads were coassembled using the metaSPAdes v3.15.3 tool (Nurk et al. 2017), and binning of the metagenome contigs was achieved by using Maxbin2 v2.2.4 (Wu et al. 2016), MetaBAT2 v1.7 (Kang et al. 2019), and CONCOCT v1.1 (Alneberg et al. 2013). The quality of the bins generated from the three tools was improved by employing the DAS Tool (Sieber et al. 2018) and assessed by the CheckM v1.0.18 tool (Parks et al. 2015). The bins with good or medium quality (completeness ≥ 50%, contamination < 10%) (Bowers et al. 2017) were retrieved and taxonomically classified using GTDB-Tk v1.7.0 (Chaumeil et al. 2020). A phylogenetic tree of the MAGs was prepared using a previously described method (Wang et al. 2023). Briefly, the universal marker genes in the MAGs were identified using the identity module of GTDB-Tk (v2.3.2) and aligned using the align module of GTDB-Tk. FastTree (v2.1) was employed to construct the phylogenetic tree of the MAGs using the concatenated universal gene alignment under the WAG + GAMMA model (Price et al. 2010). Next, the phylogenetic tree was imported into iTOL (Letunic and Bork 2016) for additional refinements. Furthermore, to assess the functional potential of the bins in the glacier ecosystem, the bins were processed using the METABOLIC v4.0 tool (Zhou et al. 2022). Additionally, the nucleotide sequences of the eight MAGs were uploaded to the RAST server to get insights into the adaptation potential of the bins using subsystem technology (Aziz et al. 2008, Overbeek et al. 2014, Brettin et al. 2015). The workflow is provided in Table S2 (Supporting Information).

## Results

### Physicochemical profile of glacial samples

The samples showed variations in physicochemical parameters across the glacial sites (Table 1). The pH of the samples was mostly consistent in the range of 6.16–6.58, the farther proglacial sample being the most acidic, while the conductivity increased linearly along the supraglacial to proglacial sites from 6.14 to 12.56 µS/cm. The analysis of solid sediment samples showed low TC (0.11%–0.19%) and S (0.05%–0.07%) and undetectable TN contents. Further elemental analysis revealed the absence of elements like Fe, K, Mn, and Zn and low concentrations of other elements ranging from 0.06 ppm (Cu) to 12.17 ppm (Ca) in the supraglacial meltwater sample. The elements Fe and K were the most abundant, followed by Mg, Mn, Ca, Zn, Cu, and P in the sediment samples of both the supraglacial and proglacial sites. No traces of heavy metals like Pb, Ni, Cr, or Cd were detected in any of the samples, depicting the pristine and uncontaminated nature of the sites.

### Bacterial community composition and diversity in the ERG

A total of 501 085 good-quality single-end sequence reads with a length of 300 nucleotides were obtained. Post denoising and chimera removal, 350 588 reads were obtained, which resulted in 622 ASVs. In contrast, paired-end sequence analysis yielded a substantially lower initial sequence reads of 49 735 resulting in 32 777 reads and 128 ASVs after denoising and chimera removal. Therefore, to avoid the risk of data loss, forward-only reads were considered.

**Figure 2.**
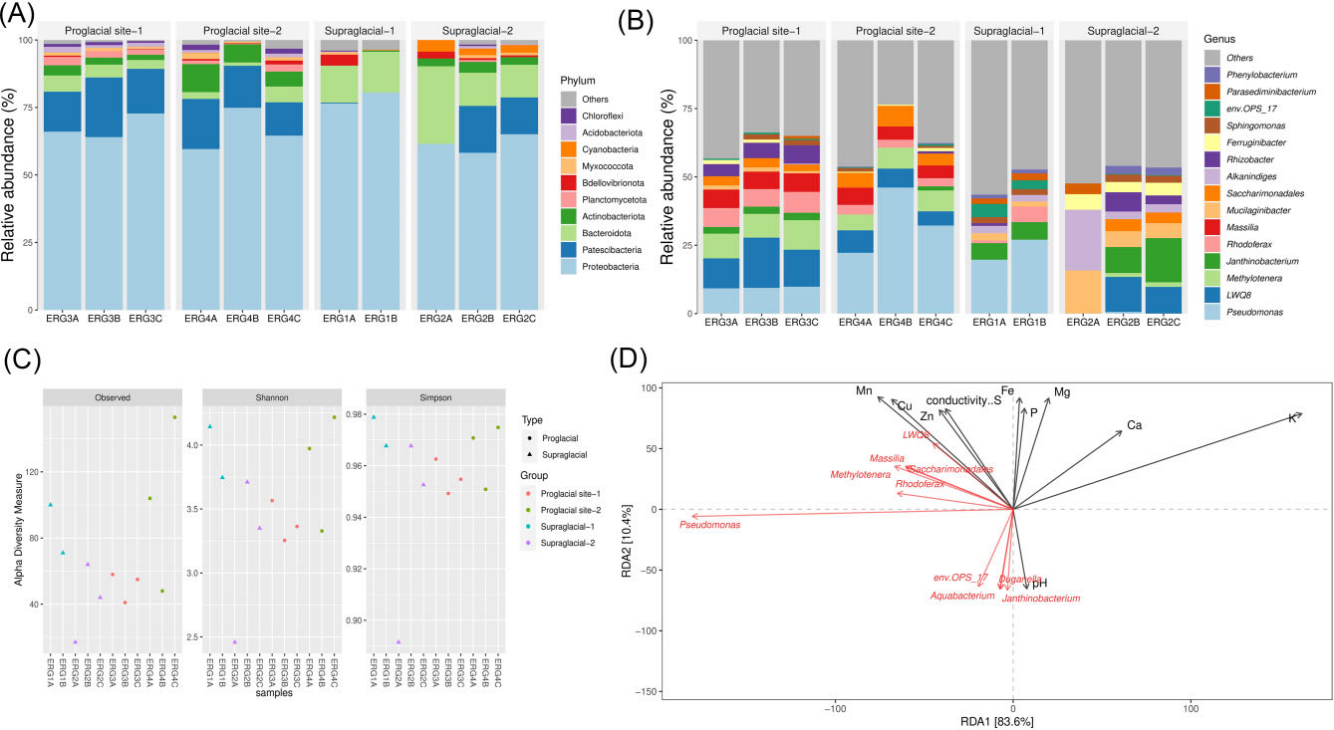
Taxonomic classification and diversity of bacterial communities across the supraglacial and proglacial samples of ERG. (A) Bacterial taxonomic composition at the phylum level. (B) Bacterial taxonomic composition at the genus level. (C) Alpha diversity indices (Observed, Shannon, and Simpson) showing the highest bacterial diversity in the farther proglacial site. (D) RDA plot indicating the correlation between the abundant bacterial genera and selective sample physicochemical parameters.

The bacterial communities resulting from the amplicon sequencing, after taxonomic classification, were assigned into ten bacterial phyla (Fig. 2A). The supraglacial meltwater sample ‘ERG1’ was dominated by the phylum Proteobacteria (76%–79%), followed by Bacteroidota (14%–15%), Bdellovibrionota (4%), and Verrucomicrobiota (1%–3%). In parallel, the supraglacial sediment samples ‘ERG2’ showed the predominance of Proteobacteria (57%–64%), accompanied by Bacteroidota (12%–27%), Patescibacteria (13%–17%), Cyanobacteria (4%–11%), and Actinobacteriota (3%–4%). The proglacial sediment samples ‘ERG3’ exhibited a prevalence of Proteobacteria (64%–73%), succeeded by Patescibacteria (15%–22%), Bacteroidota (3%–6%), Actinobacteriota (2%–4%), and Planctomycetota (2%–3%). Likewise, the proglacial samples farther from the snout ‘ERG4’ showed a significant distribution of Proteobacteria (59%–75%) and fractions of Patescibacteria (12%–18%), Actinobacteriota (5%–10%), Bacteroidota (1%–6%), Planctomycetota (1%–3%), and Chloroflexi (2%). Additionally, ERG4 showed a relatively higher presence of some other bacterial phyla like Acidobacteriota, Gemmatimonadata, Myxococcota, and Armatimonadota, though at lesser abundance (≤ 1%). At the genus level, the bacterial communities across the supraglacial and proglacial sites were majorly resolved into 15 known dominant genera (Fig. 2B). Of these, ERG1 showed a higher relative abundance of *Pseudomonas, Janthinobacterium*, and *Sphingobacteriales env.OPS 17*, while ERG2 showed the key dominance of LWQ8, *Janthinobacterium*, and *Mucilaginibacter*. Genus-wise, ERG3 and ERG4 showed higher abundances of *Pseudomonas*, LWQ8, *Methylotenera, Massilia*, and *Rhodoferax*. Additionally, ERG4 presented an elevated abundance of uncultured *Saccharimonadales*. Besides, many other known and undefined genera were detected at lower abundances (≤ 1%).

The alpha diversity indices (Shannon, Simpson, and Observed) indicated the highest diversity in the farther proglacial sample (ERG4) and the supraglacial meltwater sample (ERG1), with the observed species being highly represented in ERG4. The supraglacial sediments (ERG2) and the near-to-snout proglacial samples (ERG3) showed similar representations of the diversity indices (Fig. 2C). The Kruskal–Wallis rank sum test suggested no significant difference in the bacterial alpha diversity between the sample groups at 95% confidence level. Further, RDA was applied to assess the influence of abiotic factors in shaping the microbial community abundance and spatial distribution. Notably, the predominant *Saccharimonadales*, LWQ8, *Massilia, Methylotenera*, and *Rhodoferax* were positively correlated with EC and elemental contents of Mn, Cu, Zn, and S (Fig. 2D), illustrating the interplay between the microbes and specific environmental factors.

### Metagenome-assembled genome reconstruction from the ERG metagenomes

**Figure 3.**
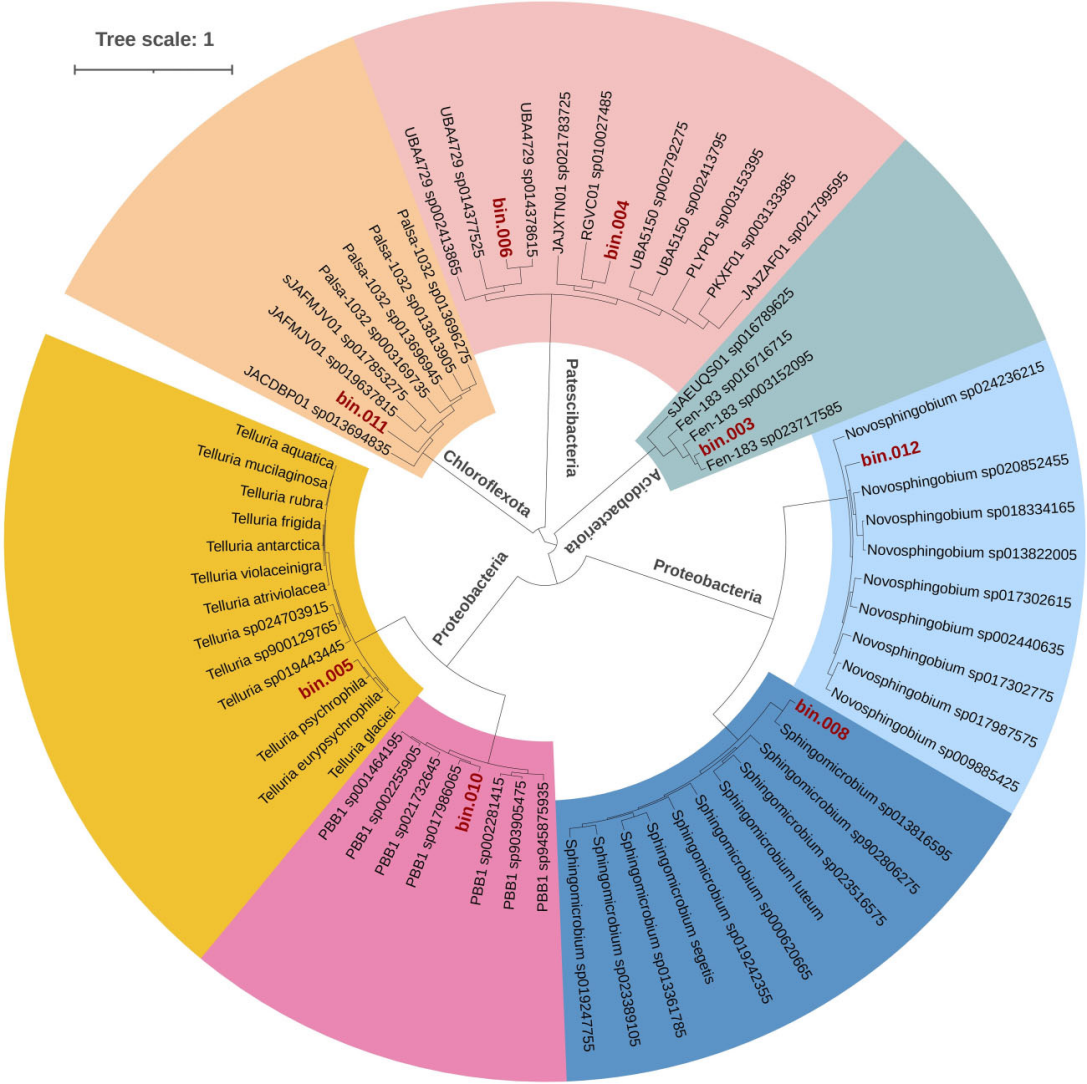
Phylogenetic tree of the eight MAGs recovered from the ERG showing affiliation to four phyla, *viz*. Acidobacteriota, Chloroflexota, Patescibacteria, and Proteobacteria. The tree was inferred based on the concatenated gene alignment of universal marker genes using the GTDB-Tk (v2.3.2). FastTree (v2.1) was employed to construct the phylogenetic tree, and iTOL was used for the final representation.

We selected two sites, namely, the supraglacial sample of the ablation zone—ERG2 and the proglacial sample farther from the glacier snout—ERG4, for metagenome sequencing and analysis. The ERG2 and ERG4 samples have 26 182 518 and 34 271 933 raw reads, respectively. After quality trimming of the reads, the ERG2 sample was left with 20 359 146 (77.8%) good-quality reads, while sample ERG4 was left with 27 226 791 (79.4%) good-quality reads. The metaSPAdes assembly consists of 21 489 contigs from both samples, with the largest contig size of 639 764 bp and N50 of 7746. A total of one high-quality (completeness > 90% and contamination < 10%) and seven medium-quality (completeness > 50% and contamination < 10%) metagenome-assembled genomes (MAGs) were retrieved from the supraglacial and proglacial metagenomes that were further analyzed. Among the eight selected MAGs, four showed affiliations with the phylum Proteobacteria (bin05, bin08, bin10, and bin12), two with Patescibacteria (bin04 and bin06), and one each with Acidobacteriota (bin03) and Chloroflexota (bin11) as revealed by GTDB-Tk analysis (Table 2). The genome statistics are provided in Table S3 (Supporting Information). The size of MAGs ranged from 0.8 to 5.4 Mb, with a GC content ranging from 46% to 68%. The Proteobacterial MAGs were further resolved into four genera, namely *Massilia, Sphingomicrobium, Rhizobacter*, and *Novosphingobium* (Fig. 3). The Patescibacterial MAGs were associated with lineages within the order Saccharimonadales (genera classified as RGVC01 and UBA4729). The remaining MAGs of Acidobacteriota and Chloroflexota corresponded to classes Thermoanaerobaculia and Ellin6529 and are classified as uncultivated genus Fen-183 and family CSP1-4, respectively.

**Table 2.** Statistics of reconstructed MAGs determined using GTDB-tk.

Bin_name	Genome size (Mb)	GC content (%)	Completeness (%)	Contamination (%)	GTDB classification
bin.03	3.4	68	92.69	3.47	p_Acidobacteriota;c_Thermoanaerobaculia;o_UBA5066;f_UBA5066;g_Fen-183
bin.04	0.8	46	63.89	2.31	p_Patescibacteria;c_Saccharimonadia;o_Saccharimonadales;f_UBA4665;g_RGVC01
bin.05	5.4	66	81.93	2.28	p_Proteobacteria;c_Gammaproteobacteria;o_Burkholderiales;f_Burkholderiaceae;g_*Massilia*
bin.06	1.1	49	66.36	1.39	p_Patescibacteria;c_Saccharimonadia;o_Saccharimonadales;f_UBA4665;g_UBA4729
bin.08	1.5	65	69.07	0.44	p_Proteobacteria;c_Alphaproteobacteria;o_Sphingomonadales;f_Sphingomonadaceae;g_*Sphingomicrobium*
bin.10	3.5	65	77.68	2.41	p_Proteobacteria;c_Gammaproteobacteria;o_Burkholderiales;f_Burkholderiaceae;g_*Rhizobacter*
bin.11	1.6	67	68.7	2.78	p_Chloroflexota;c_Ellin6529;o_CSP1-4;f_CSP1-4
bin.12	2.29	64	75.48	0.57	p_Proteobacteria;c_Alphaproteobacteria;o_Sphingomonadales;f_Sphingomonadaceae;g_*Novosphingobium*

### Metabolic functions of MAGs regulating the nutrient cycles in ERG

**Figure 4.**
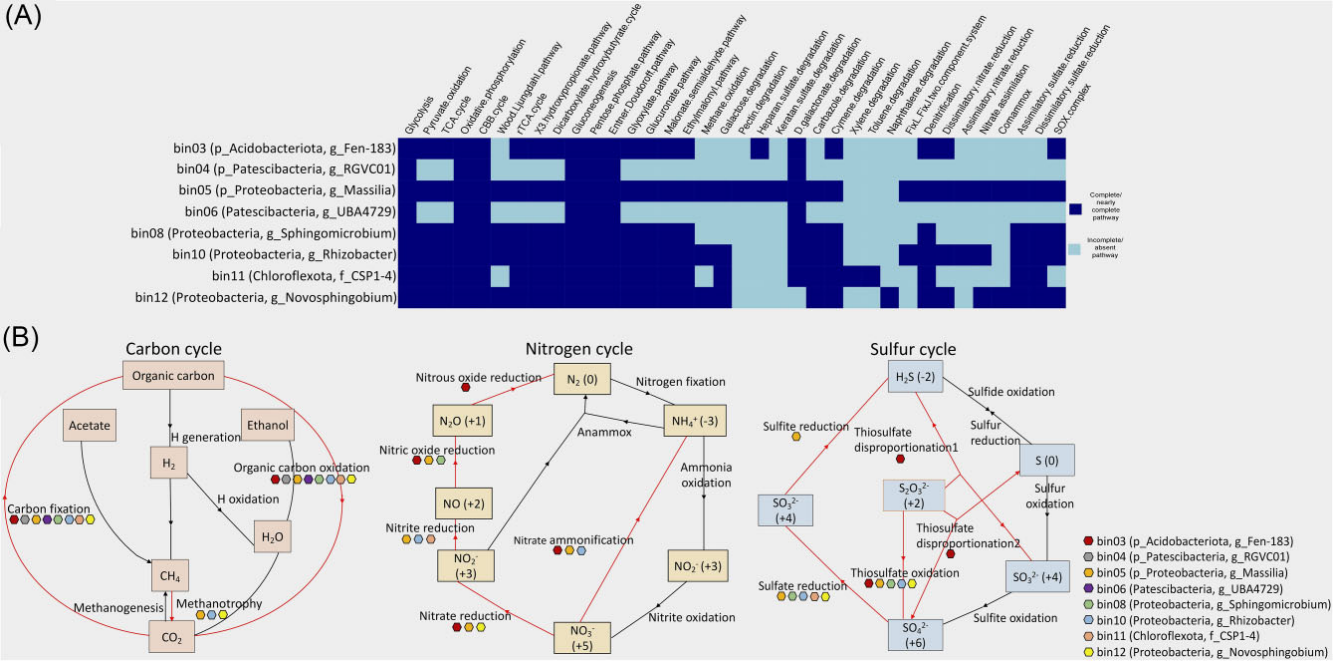
Functional analysis of eight recovered MAGs using METABOLIC v4.0 tool. (A) Heatmap representation of the selected metabolic functions in each MAG according to the KEGG module, where the dark-blue box indicates a complete or nearly complete pathway and the light-blue box indicates an incomplete or absent pathway. A nearly complete pathway indicates the presence of most of the steps in the metabolic pathway, as inferred from the detection of annotated proteins. Role of different bacterial MAGs or taxa in (B) carbon, nitrogen, and sulphur cycling in the glacial ecosystem based on the presence and absence of genes given by KEGG module step hit. The coloured boxes represent individual MAGs, red-coloured line indicates the presence, while black-coloured line indicates an absence or an incomplete pathway.

The metabolic pathways of the MAGs were deciphered using the METABOLIC tool (Table S4, Supporting Information). The tool enables the prediction of the metabolic and biogeochemical functional profiles of the bins. We found that the ERG MAGs are actively involved in the cycling of nutrients, as depicted by the KEGG module step hits (Fig. 4A and B). Aerobic respiration was predominant in all the MAGs, as evidenced by the occurrence of genes for glycolysis, tricarboxylic acid cycle, pyruvate oxidation, and oxidative phosphorylation. However, Patescibacterial MAGs were restricted to glycolysis and oxidative phosphorylation. The ability of MAGs to fix CO_2_ into organic molecules was depicted by the presence of genes for five carbon fixation pathways, i.e. Calvin–Benson–Bassham (CBB), Wood–Ljungdahl, reductive tricarboxylic acid (rTCA), 3-hydroxypropionate, and dicarboxylate-hydroxybutyrate. While the CBB pathway was well represented in all eight MAGs, Patescibacterial MAGs did not carry genes for the other four pathways. The Wood–Ljungdahl pathway that fixes CO_2_ or CO or other C1 carbon into acetyl-CoA was only exhibited by the Proteobacterial MAGs. The Proteobacterial MAGs’ potential to metabolize C1 molecules as a carbon source was reflected by the genes for methane oxidation (*mxaF/mdh1* and *mxaI/mdh2*). Additionally, Proteobacterial MAGs (*Massilia, Rhizobacter* and *Novosphingobium*) showed the potential for complex carbon degradation, as reflected by the degradation genes for pectin, galactose, heparan sulfate, and d-galactonate, while Chloroflexota MAG had the genes for aromatics degradation of toluene, xylene, cymene, and carbazole. Furthermore, the central carbohydrate metabolism processes of gluconeogenesis, pentose phosphate pathway, and Entner–Doudoroff pathway were highly represented in all eight MAGs. Besides, Acidobacteriota, Proteobacteria, and Chloroflexota MAGs showed the potential for other carbohydrate metabolism processes like the glyoxylate, glucuronate, malonate semialdehyde, and ethylmalonyl pathways.

The pathways associated with nitrogen cycling were detected in all the MAGs assigned to Proteobacteria, Acidobacteriota, and Chloroflexota, while completely absent in Patescibacteria (Fig. 4B). The MAGs retrieved from ERG metagenomes were limited to dissimilatory and assimilatory nitrate reduction and denitrification-related pathways. Notably, Acidobacteriota and Proteobacteria (*Massilia*) contained most nitrogen metabolism genes. A complete denitrification potential was observed in these MAGs by encoding periplasmic nitrate reductase NapAB, nitrate reductase NarGHI, nitrite reductase NirK and NirS, nitric oxide reductase NorBC, CYP55, and nitrous oxide reductase NosZ. Similarly, the two MAGs showed full potential for dissimilatory nitrate reduction as they contained nitrate reductase NapAB, NarGHI, and nitrite reductase NirBD, NrfAH. Only *Massilia* and *Rhizobacter* carried partial genes for assimilatory nitrate reduction to ammonia (NarB, NR, and NasAB). None of the MAGs showed the capacity for nitrogen fixation.

The sulphur cycling pathway was absent in Patescibacterial MAG, while it was highly represented in the Proteobacterial MAG of *Massilia* (Fig. 4B). Chiefly, *Massilia* showed the capacity for complete assimilatory sulfate reduction (CysND, Sat, PAPSS, CysC, CysH, CysJI, and Sir), while other Proteobacteria and Chloroflexota MAGs showed partial assimilatory and dissimilatory sulfate reduction (CysND and Sat) potential. Moreover, a complete sulphur oxidation (SOX) enzyme complex (SoxA, SoxX, SoxB, SoxC, SoxY, and SoxZ) was observed in the Acidobacteriota and Proteobacterial MAGs. The SOX complex is capable of oxidizing thiosulfate, sulfite, sulfide, or elemental sulphur to sulfate (Wang et al. 2019).

### Adaptation of ERG MAGs to environmental stress

RAST analysis of the MAGs for proteins involved in stress response revealed their distribution into categories like DNA repair, oxidative stress, osmotic stress, periplasmic stress, protein chaperones, and carbohydrate starvation (Table S5, Supporting Information). For DNA repair against UV or desiccation, genes encoding nucleotide exchange repair proteins, i.e. UvrABC excinuclease, were identified in the MAGs except for *Sphingomicrobium* and *Novosphingobium*. Mismatch repair proteins MutL–MutS and nonhomologous end-joining repair genes (*ligC, ligD*, and ku domain protein) were detected in Acidobacteriota, Patescibacteria (g_UBA4729), and Proteobacteria (*Massilia, Sphingomicrobium*, and *Novosphingobium*). Additionally, homologous repair genes (*recFOR*) were present in Patescibacteria (g_RGVC01) and Proteobacteria MAGs. Many oxidative stress response proteins, including superoxide dismutase and ruberyththrin/rubredoxin, were present in a majority of the MAGs. Besides, glutathione reductase and glutathione peroxidase for the redox cycle and glutathione S-transferase and lactoylglutathione lyase for nonredox reactions were identified in the Proteobacterial MAGs. In response to osmotic stress, the MAGs of Acidobacteriota, Patescibacteria (g_UBA4729), and Proteobacteria (*Massilia, Sphingomicrobium*, and *Rhizobacter*) were equipped with cyclic beta-1,2-glucan synthase for the synthesis of osmoregulated periplasmic glucans. Besides, choline and betaine uptake and betaine biosynthesis proteins, including sarcosine oxidase, choline dehydrogenase, and choline uptake protein BetT, were detected in *Novosphingobium*. Proteins for combating periplasmic stress were also identified, chiefly the HtrA protease, which has both chaperone and proteolytic activities for removing misfolded proteins. Others, like outer membrane stress sensor protease DegS and outer membrane protein H precursor, were present in Proteobacteria (*Massilia* and *Rhizobacter*). Excluding Chloroflexota, all MAGs possessed the key chaperone proteins DnaJ and DnaK. Moreover, the genomes of Acidobacteriota and Patescibacteria carried the genes for carbon starvation protein A, which is known to be a pyruvate transporter (Gasperotti et al. 2020).

## Discussion

Microorganisms are an integral part of the biosphere that drives the ecological processes through the global biogeochemical cycling of nutrients. Glaciers and ice sheets, as cryosphere components, alone cover ∼10% of the Earth’s surface (IPCC 2019). Being extremely sensitive to the warming climate, the accelerated retreat of alpine glaciers and ice masses would undoubtedly perturb the local biome and the associated ecosystem processes. Against this backdrop, the cryospheric microbiome is of much significance yet remains one of the most cryptic and poorly characterized among other microbiomes (Bourquin et al. 2022). To further our understanding of the alpine microbial communities and their roles in ecosystem functioning, our study investigates the transect along an ablation zone supraglacial and nearby proglacial site of a vulnerable Himalayan glacier.

A glacier ecosystem includes supraglacial and proglacial habitats with varying physicochemical properties. Our microbiome analysis of samples on the ablation zone of ERG revealed many shared bacterial phyla, varying only in relative abundance. The dominant phyla, Proteobacteria and Bacteroidota, observed in the supraglacial ice meltwater samples are predominant in the ice samples collected from other glaciers as well (Wilhelm et al. 2013, Lutz et al. 2015, Garcia-Lopez et al. 2019). The prevalent Patescibacteria in supraglacial and proglacial sediment samples constitute an uncharacterized group frequently documented in cold environments (Kumar et al. 2022, Rathore et al. 2022). Cyanobacteria, identified as the main primary producers in supraglacial habitats worldwide (Anesio et al. 2017, Rathore et al. 2022, Jaarsma et al. 2023), was detected in the ERG surface sediments. Interestingly, Bdellovibrionota—a predatory bacterial group, occured in the supraglacial meltwater samples. Previously, this bacteria has been reported from Antarctic soils, marine waters, and perialpine lakes (Paix et al. 2019, Li et al. 2021, Ortiz et al. 2021). The predominance of the genus *Pseudomonas* in the supraglacial meltwater corroborates our previous culture-dependent study (Mukhia et al. 2022b). The uncultured genus *Saccharimonadales* LWQ8 was more enriched in the sediment samples of supraglacial and proglacial sites. As anticipated, the proglacial site farther from the snout showed the highest bacterial alpha diversity as it is a comparatively older soil to have developed after the glacial retreat featuring greater microbial activities for bioweathering. The supraglacial meltwater sample showed the next highest alpha diversity. The surface ice is subject to the invasion of minerals and microbial cells from the wind, while the ice-meltwater provides water for microbial growth and metabolism that together facilitates microbial colonization in this zone (Anesio and Laybourn-Parry 2012). Ecologists often use the concept of the source-sink hypothesis to estimate the flow of microorganisms between habitats that might explain the shaping of observed differences (Burns et al. 2016). Source-sink dynamics could be pivotal in shaping the observed community dynamics within a studied ecosystem (Ezzat et al. 2022, Rolli et al. 2022). In this context, supraglacial habitats may serve as diversity sources, supplying microorganisms to proglacial sink habitats. Subsequently, the differential environmental conditions, nutrient availability, and physical characteristics between source and sink habitats facilitate the unique establishments, impacting the entire ecosystem dynamics. To unveil the inclusive intricacies of the glacial bacterial communities, we delved into the metagenomes of representative supraglacial and proglacial samples. The observed phyla of the reconstructed ERG MAGs, namely Proteobacteria, Patescibacteria, Acidobacteriota, and Chloroflexota, are often recovered from other glaciers and permafrosts as well (Xue et al. 2019, 2020, Varliero et al. 2021, Busi et al. 2022). Affirming our findings of the amplicon data, we retrieved two MAGs belonging to the order Saccharimonadales and one each of the genus *Massilia* and *Rhizobacter*. Fascinatingly, the MAG within Acidobacteriota was annotated to the class Thermoanaerobaculia, which consists of thermophilic bacteria previously isolated exclusively from thermal habitats (Dedysh and Lawson 2020). The phylum Chloroflexota was observed in both amplicon and metagenome samples and the retrieved MAG was taxonomically labeled up to the family level as CSP1-4. The sequences in this clade mostly originate from soil or river sediments as per SILVA taxonomy (Mehrshad et al. 2018).

The geographical location of the high-altitude Himalayan glaciers represents a hostile environment for life to flourish. How microorganisms adapt to such extreme conditions and perform metabolic activities driving the ecosystem is indeed intriguing. As glacier ecology is primarily driven by microbial metabolism, elucidation of metabolic pathways is key to demonstrating the role of bacterial communities in niche-specific biogeochemical cycles. Our metagenomic investigation of MAGs focused on unraveling the key metabolic strategies adopted to meet energy and carbon requirements. To no surprise, all the identified MAGs showed great capacity for aerobic respiration, which highlights the preferred mode of energy fulfillment in the ERG communities. None of the MAGs demonstrated any noticeable ability for fermentation. Such a finding was in concurrence with other reports from Arctic and Antarctic soils (Xue et al. 2020, Ortiz et al. 2021). Physiological analysis revealed a shallow organic carbon content of 0.11%–0.19% in the ablation zone of the ERG region. Concurrently, we observed all the major carbon fixation pathways in the recovered MAGs, particularly in Proteobacteria. Consequently, carbon dioxide fixation seems to be the dominant carbon uptake and metabolism mode instead of organic carbon oxidation in the nutrient-limited glacial environment. The prevalence of diverse CO_2_-fixation pathways in the glacial MAGs provides evidence for efficient autotrophic potential. Mainly, three MAGs (*Massilia, Sphingomicrobium*, and *Rhizobacter*) within Proteobacteria showed an almost complete CBB cycle, including the central enzyme RuBisCO, which suggests the dominance of this pathway among all. Another glacier ecosystem has gained a similar interpretation (Trivedi et al. 2020). The next common carbon fixation pathway in the ERG may be the reductive TCA cycle, as the genes for the pathway were also nearly complete in Acidobacteriota and Proteobacteria (*Rhizobacter* and *Novosphingobium*) MAGs. The other carbon metabolism pathways in the MAGs were mostly partial and may be secondary processes for carbon incorporation.

As interpreted from the results, dissimilatory nitrate reduction and denitrification may be the dominant nitrogen metabolism pathways in the recovered MAGs. The findings were similar to those observed in other Arctic glacier MAGs (Trivedi et al. 2020, Tian et al. 2022). While dissimilatory nitrate reduction to ammonium is a means of energy conservation that retains the N in the system for crucial biological processes, denitrification releases N back into the atmosphere. Both functions are essential for maintaining the nitrogen dynamics in an ecosystem. Most denitrifiers are known to switch from aerobic to anaerobic respiration under O_2_-limited conditions. The glacier surface and forefield at the ablation zone are subject to changes in aerobic and anaerobic conditions. This occurs due to intense environmental fluctuations consisting of heavy snow and rainfall followed by dry periods of vigorous light intensity (Tian et al. 2022). The complete dissimilatory nitrate-reducing and denitrifying genes in some of the MAGs suggest their potential for an anaerobic mode of respiration to better adapt to the changing environmental conditions. Denitrification is active in many other alpine glaciers (Chen et al. 2022, Murakami et al. 2022). However, N fixation was not detected in the MAGs, as was observed in the Canadian High Arctic permafrost and Svalbard forefield MAGs (Wu et al. 2021, Tian et al. 2022), which might suggest the minimal contribution of the process in the nitrogen cycle. Although, the FixL–FixJ two-component regulatory system was identified in the MAGs of Proteobacteria (*Massilia* and *Rhizobacter*) that stimulate nitrogen fixation under low oxygen conditions.

Sulphur metabolism pathways in the MAGs were dominated by assimilatory sulfate reduction and SOX, as suggested by the presence of complete pathway genes. Assimilatory sulfate reduction results in the biosynthesis of sulphur-containing amino acids in anoxygenic bacteria. The snow melting during summers enhances the weathering of rocks that contribute to sulfate content, which is actively metabolized by the chemolithotrophs (Rathore et al. 2022). Hence, ERG MAGs are better involved in the utilization of inorganic sulphur for biosynthesis processes. Likewise, SOX is a vital aspect of biogeochemical sulphur cycling, where thiosulfate oxidation leads to the formation of elemental sulphur or sulfate. We found that the Acidobacteriota and Proteobacteria MAGs showed the full potential for this means of energy gain. Gammaproteobacteria MAGs have been reported for similar activity in a Canadian High Arctic permafrost (Wu et al. 2021). Some of the MAGs carried partial genes for dissimilatory sulfate reduction, which indicates the rarity of this pathway but might aid in the bioweathering and release of nutrients for sustaining the sulphur cycle. Among all MAGs, Patescibacteria contained the least metabolism pathways that may be attributed to the obligately symbiotic lifestyle of the phylum and, hence, is deficient in alternative pathways for carbon acquisition or energy conservation (Ortiz et al. 2021).

To get insights into the basis of microbial life in ERG, we checked the stress response genes in the supraglacial and proglacial MAGs. Besides low-temperature conditions, the microorganisms must survive through intense UV irradiations and oxidative stress, desiccation, low water activity, osmotic stress, and low nutrient availability in high-altitude glaciers (Collins and Margesin 2019). We observed that the most prevalent genes were associated with DNA repair and oxidative stress response. DNA repair genes are crucial for microbial survival against UV and desiccation stress (Liu et al. 2022). Consequently, we detected *uvrABC, mutS, mutL, lexA, ligC*, and *ligD*, and other repair proteins like *radA, radC, recA, recN, recF, recO*, and *recR*. Low environmental temperature is often linked with high oxidative stress (Chattopadhyay et al. 2011). Most MAGs carried superoxide dismutase, which is a key antioxidant enzyme for oxidative stress tolerance in aerobic organisms. The enrichment of glutathione S-transferase, known to quench reactive molecules, signifies the efficient regulation of oxidative stress in glacier bacteria, protecting the cells from oxidative burst and harmful effects of UV radiation (Kumar and Trivedi 2018). For osmotic stress adaptation, cyclic beta-1,2-glucan synthase particularly occurred in most MAGs. This enzyme is responsible for synthesizing a group of osmoregulated periplasmic glucans involved in osmoadaptation (Bontemps-Gallo et al. 2017). We found that the Proteobacteria MAGs are well-equipped with periplasmic stress sensor proteins like *degS* and *ompH* since the microbial cell envelope is the first barrier and primary defense against environmental assaults. The molecular chaperone proteins *dnaJ* and *dnaK* that facilitate bacterial survival in a stressful environment (Mukhia et al. 2022a) occurred uniformly in the glacial MAGs.

## Conclusions

With rapidly changing ecosystems, the microbial dynamics of the supraglacial sites and immediate proglacial sites need to be understood as the glacial retreat influences the nutrient fluxes at the catchment sites, which later develop into a new ecosystem. To our knowledge, this is the first bacterial metagenomic assessment of the ERG. The glacial sites showed major dominance of bacterial phyla Proteobacteria, Bacteroidetes, and Patescibacteria. The observation of diverse adaptive and metabolic traits in the recovered MAGs demonstrates the persistence of the resident microflora for successful colonization in the oligotrophic stressful environment. The presence of multiple carbon fixation pathways indicates the significance of chemolithoautotrophic Proteobacteria in the ecosystem. Aerobic respiration and nitrogen and sulphur metabolism potential were prevalent in the MAGs. This signifies their metabolic flexibility for perseverance and energy conservation, contributing collectively to the ecosystem functioning. Altogether, the detected nutrient cycling pathways add to our knowledge of the roles of bacteria in global geochemical fluxes.

## Supplementary Material

fiae012_Supplemental_Files

## Data Availability

The SRA files of 16S rRNA gene amplicon sequences have been deposited in the GenBank database with BioSample accessions (SAMN30684607-SAMN30684615 and SAMN30684629-SAMN30684630). The shotgun sequences are deposited under the BioProject PRJNA932945 in NCBI. MAGs are deposited under BioProject PRJNA933897 and PRJNA933900 with genome accessions: bin03 (JARXKK000000000), bin04 (JARXKL000000000), bin05 (JARXKM000000000), bin06 (JARXKN000000000), bin08 (JARXKO000000000), bin10 (JARXKP000000000), bin11 (JARXKQ000000000), and bin12 (JARXKR000000000). The raw amplicon sequences and analysis files, MAG sequences obtained from the KBase server, and analysis codes are uploaded to the GitHub repository at https://github.com/anilchauhanhp9/Amplicons-Metagenome-data-analysis.git.
