# Supplementary material for: Bacterial community distribution and functional potentials provide key insights into their role in the ecosystem functioning of a retreating Eastern Himalayan glacier
Source: FEMS Microbiol Ecol. 2024 Feb 1;100(3):fiae012. doi: 10.1093/femsec/fiae012 (PMC10876117; doi:10.1093/femsec/fiae012)
Supplement: fiae012_Supplemental_Files [file fiae012_supplemental_files.zip › Table S1.docx]

**Table S1.** Statistics of the number of input sequences and sequences left after DADA2 filtration in each sample.

| **Sample-id** | **Input reads** | **Filtered reads** | **Input reads that passed filter (%)** | **Denoised reads** | **Non-chimeric reads** | **Input non-chimeric reads (%)** |
| --- | --- | --- | --- | --- | --- | --- |
| ERG1A | 180709 | 60999 | 33.76 | 55334 | 52510 | 29.06 |
| ERG1B | 173920 | 39508 | 22.72 | 31864 | 31168 | 17.92 |
| ERG2A | 57659 | 7021 | 12.18 | 4915 | 4627 | 8.02 |
| ERG2B | 134893 | 24805 | 18.39 | 21957 | 20049 | 14.86 |
| ERG2C | 99507 | 20333 | 20.43 | 16669 | 15401 | 15.48 |
| ERG3A | 94859 | 21534 | 22.7 | 17551 | 16289 | 17.17 |
| ERG3B | 94837 | 18137 | 19.12 | 13936 | 13936 | 14.69 |
| ERG3C | 113465 | 21336 | 18.8 | 17437 | 16787 | 14.79 |
| ERG4A | 121708 | 94326 | 77.5 | 77278 | 58597 | 48.15 |
| ERG4B | 71402 | 53677 | 75.18 | 46533 | 28003 | 39.22 |
| ERG4C | 185087 | 139409 | 75.32 | 120920 | 93221 | 50.37 |
