# Supplementary material for: Bacterial community distribution and functional potentials provide key insights into their role in the ecosystem functioning of a retreating Eastern Himalayan glacier
Source: FEMS Microbiol Ecol. 2024 Feb 1;100(3):fiae012. doi: 10.1093/femsec/fiae012 (PMC10876117; doi:10.1093/femsec/fiae012)
Supplement: fiae012_Supplemental_Files [file fiae012_supplemental_files.zip › Table S2.docx]

**Table S2.** Data processing codes and workflow used in the manuscript.

**## Running Qiime-2**

conda activate qiime2-2021.2

qiime tools import \

--type 'SampleData[PairedEndSequencesWithQuality]' \

--input-path . \

--input-format CasavaOneEightSingleLanePerSampleDirFmt \

--output-path demux-paired-end.qza

qiime demux summarize \

--i-data demux-paired-end.qza \

--o-visualization demux.qzv

qiime dada2 denoise-single \

--i-demultiplexed-seqs demux-paired-end.qza \

--p-trim-left 0 \

--p-trunc-len 300 \

--p-max-ee 5.0 \

--o-representative-sequences rep-seqs.qza \

--o-table table.qza \

--p-n-threads 0 \

--o-denoising-stats denoising-stats.qza –verbose

qiime feature-table summarize \

--i-table table.qza \

--o-visualization table.qzv \

--m-sample-metadata-file sample-metadata.tsv

qiime feature-table tabulate-seqs \

--i-data rep-seqs.qza \

--o-visualization rep-seqs.qzv

qiime metadata tabulate \

--m-input-file denoising-stats.qza \

--o-visualization denoising-stats.qzv

qiime metadata tabulate \

--m-input-file table.qza \

--o-visualization otu_table_per_sample.qzv

qiime phylogeny align-to-tree-mafft-fasttree \

--i-sequences rep-seqs.qza \

--o-alignment aligned-rep-seqs.qza \

--o-masked-alignment masked-aligned-rep-seqs.qza \

--o-tree unrooted-tree.qza \

--o-rooted-tree rooted-tree.qza

qiime diversity core-metrics-phylogenetic \

--i-phylogeny rooted-tree.qza \

--i-table table.qza \

--p-sampling-depth 4626 \

--m-metadata-file sample-metadata.tsv \

--output-dir core-metrics-results

qiime diversity alpha-rarefaction \

--i-table table.qza \

--i-phylogeny rooted-tree.qza \

--p-max-depth 20049 \

--m-metadata-file sample-metadata.tsv \

--o-visualization alpha-rarefaction.qzv

qiime feature-classifier classify-sklearn \

--i-classifier silva-138-99-nb-classifier.qza \

--i-reads rep-seqs.qza \

--output-dir silva/taxonomy.qza

qiime metadata tabulate \

--m-input-file silva/taxonomy.qza/classification.qza \

--output-dir silva/taxonomy.qzv

qiime taxa barplot \

--i-table table.qza \

--i-taxonomy silva/taxonomy.qza/classification.qza \

--m-metadata-file sample-metadata.tsv \

--output-dir silva/taxa-bar-plots.qzv

**## input for Phyloseq-R**

qiime tools export --input-path rarefied_table.qza --output-path .

biom convert --to-tsv -i feature-table.biom -o rarefied-feature-table.tsv

qiime tools export --input-path silva-taxonomy.qza --output-path .

qiime tools export --input-path rooted-tree.qza --output-path .

**## Phyloseq R**

library("phyloseq")

library("ggplot2")

library("readxl")

library("plyr")

library("dplyr")

library("tibble")

library("microbiomeSeq")

library("vegan")

library("ape")

library("dunn.test")

dir()

otu_table <- read.table("rarefied-feature-table.tsv",

sep = "\t", header = TRUE, row.names = 1)

class(otu_table)

head(otu_table)

otu_table <- as.matrix(otu_table)

class(otu_table)

taxa <- read.table("silva-taxonomy.tsv",

sep = "\t", header = TRUE, row.names = 1)

taxa <- as.matrix(taxa)

head(taxa)

class(taxa)

metadata <- read.table("sample-metadata.tsv",

sep = "\t", header = TRUE, row.names = 1)

head(metadata)

trefile = read.tree(file = "tree.nwk")

print(trefile)

OTU <- otu_table(otu_table, taxa_are_rows=TRUE)

TAX = tax_table(taxa)

sample = sample_data(metadata)

phyloseq_obj <- phyloseq(OTU, TAX, sample)

print(phyloseq_obj)

plot_richness(phyloseq_obj, measures=c("Shannon", "simpson", "Observed" ),

x="samples", color="Group", shape="Type")

**## Kruskal wallis test**

alpha_Supraglacial1 <- subset_samples(phyloseq_obj, Group=="Supraglacial-1")

alpha_Supraglacial2 <- subset_samples(phyloseq_obj, Group=="Supraglacial-2")

alpha_Proglacial3 <- subset_samples(phyloseq_obj, Group=="Proglacial-3")

alpha_Proglacial4 <- subset_samples(phyloseq_obj, Group=="Proglacial-4")

all_alpha <- merge_phyloseq(alpha_Supraglacial1, alpha_Supraglacial2,

alpha_Proglacial3, alpha_Proglacial4 )

all_alpha

alpha_observed <- estimate_richness(all_alpha, measures = "Observed")

alpha_Shannon <- estimate_richness(all_alpha, measures = "Shannon")

alpha_simpson <- estimate_richness(all_alpha, measures = "simpson")

alpha.stats <- cbind(alpha_observed, sample_data(all_alpha))

kruskal.test(Observed~Group, alpha.stats)

dunn.test(alpha.stats$Observed, alpha.stats$Group,

method = "bonferroni")

alpha.stats <- cbind(alpha_Shannon, sample_data(all_alpha))

kruskal.test(Shannon~Group, alpha.stats)

dunn.test(alpha.stats$Shannon, alpha.stats$Group,

method = "bonferroni")

alpha.stats <- cbind(alpha_simpson, sample_data(all_alpha))

kruskal.test(Simpson~Group, alpha.stats)

dunn.test(alpha.stats$simpson, alpha.stats$Group,

method = "bonferroni")

**## Running Metabolic tool**

## The process of preprocessing, trimming, MAG generation and GTDB classification were done in the KBase server according to a previously described methodology (<https://doi.org/10.1038/s41596-022-00747-x>). The MAG sequences obtained from KBase server were used for METABOLIC analysis.

conda activate metabolic_v4.0

perl ./METABOLIC-G.pl -in-gn ./bins -t 20 -o ./metabolic_G_out

**## Metagenome assembled genome (MAGs) phylogenetic tree**

conda activate gtdbtk-2.3.2

gtdbtk identify --genome_dir [genomes] --out_dir [identify_output] --cpus 16

gtdbtk align --identify_dir [identify_output] --out_dir [align_output] --cpus 16

FastTree alignment.file -wag -gamma > tree_file
