# Supplementary material for: Bacterial community distribution and functional potentials provide key insights into their role in the ecosystem functioning of a retreating Eastern Himalayan glacier
Source: FEMS Microbiol Ecol. 2024 Feb 1;100(3):fiae012. doi: 10.1093/femsec/fiae012 (PMC10876117; doi:10.1093/femsec/fiae012)
Supplement: fiae012_Supplemental_Files [file fiae012_supplemental_files.zip › Table S3_Supplementary data_R1.pdf]

Table S3: The number of contigs, rRNA, tRNA, CDS and N50 value of each Metagenome assembled genome (MAG) recovered from the East Rathong Glacier metagenomes.

Bin name	Contigs	N50 (Kb)	rRNA	tRNA	CDS
bin.03	304	19.409	-	37	3159
bin.04	29	49.942	1 (16S)	36	906
bin.05	691	11.506	-	34	5067
bin.06	25	639.764	1, 2 (5S, 23S)	41	1084
bin.08	287	6.996	-	24	1774
bin.10	419	10.876	-	24	3533
bin.11	321	5.453	1, 1, 1 (5S, 16S, 23S)	22	1818
bin.12	363	9.507	-	35	2427
