# Supplementary material for: Bacterial community distribution and functional potentials provide key insights into their role in the ecosystem functioning of a retreating Eastern Himalayan glacier
Source: FEMS Microbiol Ecol. 2024 Feb 1;100(3):fiae012. doi: 10.1093/femsec/fiae012 (PMC10876117; doi:10.1093/femsec/fiae012)
Supplement: fiae012_Supplemental_Files [file fiae012_supplemental_files.zip › Table S5_Supplementary data_R1.pdf]

**Supplementary Table S5.** Genes encoding proteins with roles in stress adaptation in different copy numbers in the glacial MAGs predicted by RAST analysis.

| Stress category | Predicted proteins | bin03 | bin04 | bin05 | bin06 | bin08 | bin10 | bin11 | bin12 |
|---|---|---|---|---|---|---|---|---|---|
| **DNA repair** | A/G-specific adenine glycosylase | 1 | 1 | 1 | 1 | 1 | 1 | | 1 |
| | Excinuclease ABC subunit A | 2 | 2 | 1 | 2 | | 1 | 1 | |
| | Excinuclease ABC subunit B | 1 | 1 | 2 | 1 | | 2 | | |
| | Excinuclease ABC subunit C | 1 | 1 | 1 | 1 | | 2 | | |
| | DNA mismatch repair protein MutL | 1 | | 1 | 1 | 1 | | | 1 |
| | DNA mismatch repair protein MutS | 1 | | 1 | 1 | 1 | | | 2 |
| | Recombination inhibitory protein MutS2 | 1 | | | 1 | | | | |
| | ATP-dependent DNA ligase LigC | | | 1 | | 3 | | | 1 |
| | ATP-dependent DNA ligase clustered with Ku protein, LigD | 2 | | 1 | 2 | 1 | | | 1 |
| | Ku domain protein | 1 | | 1 | 1 | 1 | | | 1 |
| | SOS-response repressor and protease LexA | 1 | | 1 | 1 | 1 | | 1 | 1 |
| | Deoxyribodipyrimidine photolyase | 1 | 1 | 1 | 1 | | 1 | | 1 |
| | DinG family ATP-dependent helicase | 1 | | 2 | 1 | | 2 | | |
| | DNA repair protein RadA | 1 | 1 | | 1 | 2 | 1 | 1 | 1 |
| | DNA repair protein RadC | | | | | 2 | | | |
| | RecA protein | 1 | 1 | 1 | 1 | 1 | | 2 | 1 |
| | DNA repair protein RecN | 1 | | 1 | 1 | 1 | 1 | | |
| | DNA recombination and repair protein RecF | | 1 | | | 1 | | | 1 |
| | DNA recombination and repair protein RecO | | 1 | 1 | | 1 | 1 | | 1 |
| | Recombination protein RecR | | 1 | 1 | | 1 | 1 | | 1 |
| | ATP-dependent DNA helicase RecQ | | | 2 | | 1 | 1 | | 1 |
| | DinG family ATP-dependent helicase YoaA | | | 1 | | 1 | 1 | | 1 |
| | DNA recombination protein RmuC | | | 1 | | 2 | 1 | | 1 |
| | DNA-3-methyladenine glycosylase | | | 1 | | | | | |
| | DNA-3-methyladenine glycosylase II | | | 2 | | | | | |
| | Formamidopyrimidine-DNA glycosylase | | | 1 | | | | | |

| | | | | | | | | | |
|---|---|---|---|---|---|---|---|---|---|
| | | | | | | | | | |
| **Oxidative stress** | Superoxide dismutase [Fe] | 2 | | 1 | 2 | | 2 | | 1 |
| | Superoxide dismutase [Mn] | 1 | 1 | | 1 | | | | |
| | Glutathione S-transferase | | | 7 | | 3 | 4 | | 7 |
| | Glutathione S-transferase family protein | | | 1 | | 1 | 1 | | 1 |
| | Lactoylglutathione lyase | | | 1 | | 1 | 2 | | 1 |
| | Glutathione peroxidase | | | 2 | | | 3 | | 1 |
| | Glutathione synthetase | | | 1 | | 1 | 1 | | 2 |
| | Glutathione reductase | | | | | 1 | 1 | | 1 |
| | Alkyl hydroperoxide reductase subunit C-like protein | 2 | | | 2 | | | | 1 |
| | Rubrerythrin/Rubredoxin | 1 | | 1 | 1 | | 1 | | |
| | Ferric uptake regulation protein FUR | 1 | | 1 | 1 | | | | 1 |
| | Fe-S oxidoreductase-like protein in Rubrerythrin cluster | 1 | | | 1 | | | | |
| | Alkyl hydroperoxide reductase subunit C-like protein | | | 1 | | 2 | | | |
| | Glutaredoxin-related protein | | | 1 | | | 1 | | |
| | Organic hydroperoxide resistance protein | | | 1 | | | | | |
| | Organic hydroperoxide resistance transcriptional regulator | | | 1 | | | | | |
| | Glutaredoxin 3 (Grx3) | | | 1 | | | 1 | | 1 |
| | Phytochrome, two-component sensor histidine kinase | | | 1 | | | | | |
| | | | | | | | | | |
| **Osmotic stress** | Cyclic beta-1,2-glucan synthase | 2 | | 2 | 2 | 2 | 1 | | |
| | Outer membrane protein A precursor | | | 3 | | | 1 | | 1 |
| | Aquaporin Z | | | 1 | | | 1 | 1 | 2 |
| | Glucans biosynthesis protein D precursor | | | | | | 1 | | |
| | Sarcosine oxidase alpha subunit | | | | | | | | 3 |
| | Sarcosine oxidase beta subunit | | | | | | | | 2 |

| | | | | | | | | | |
|---|---|---|---|---|---|---|---|---|---|
| | Sarcosine oxidase delta subunit | | | | | | | | 2 |
| | Choline dehydrogenase | | | | | | | | 1 |
| | High-affinity choline uptake protein BetT | | | | | | | | 1 |
| | | | | | | | | | |
| **Periplasmic Stress** | HtrA protease/chaperone protein | 1 | | 1 | 1 | 1 | 1 | | |
| | Outer membrane protein H precursor | | | 1 | | | 1 | | |
| | Outer membrane stress sensor protease DegS | | | 1 | | | 1 | | |
| | Sigma factor RpoE negative regulatory protein RseB precursor | | | | | | 1 | | |
| | | | | | | | | | |
| **Protein chaperones** | Chaperone protein DnaJ | 3 | 1 | 2 | 3 | 1 | 2 | | 1 |
| | Chaperone protein DnaK | 3 | 1 | 2 | 3 | 1 | 1 | | 2 |
| | Chaperone protein HtpG | | | 1 | | | 1 | | |
| | Chaperone protein HscA | | | 1 | | | 1 | | |
| | Chaperone protein HscB | | | 1 | | | 1 | | |
| | DnaJ-class molecular chaperone CbpA | | | 2 | | 1 | 2 | | 1 |
| | Heat shock protein GrpE | 1 | 1 | 1 | 1 | 1 | | | 1 |
| | HspR, transcriptional repressor of DnaK operon | 1 | | | 1 | | | | |
| | | | | | | | | | |
| **Carbon Starvation** | Carbon starvation protein A | 1 | | | 1 | | | | |
