# Supplementary material for: Bacterial community distribution and functional potentials provide key insights into their role in the ecosystem functioning of a retreating Eastern Himalayan glacier
Source: FEMS Microbiol Ecol. 2024 Feb 1;100(3):fiae012. doi: 10.1093/femsec/fiae012 (PMC10876117; doi:10.1093/femsec/fiae012)
Supplement: fiae012_Supplemental_Files [file fiae012_supplemental_files.zip › File Legends_Supplementary data_R1.docx]

**Supplementary files**

**Table S1.** Statistics of the number of input sequences and sequences left after DADA2 filtration in each sample.

**Table S2.** Data processing codes and workflow used in the manuscript.

**Table S3.** The number of contigs, rRNA, tRNA, CDS and N50 value of each Metagenome assembled genome (MAG) recovered from the East Rathong Glacier (ERG) metagenomes.

**Table S4.** Selected KEGG modules and the associated pathways identified in the eight MAGs retrieved from the ERG metagenomes.

**Table S5.** Genes encoding proteins with roles in stress adaptation in different copy numbers in the glacial MAGs predicted by RAST analysis.
